# Supplementary material for: Reduced Cholinergic Activity in the Hippocampus of Hippocampal Cholinergic Neurostimulating Peptide Precursor Protein Knockout Mice
Source: Int J Mol Sci. 2019 Oct 28;20(21):5367. doi: 10.3390/ijms20215367 (PMC6862429; doi:10.3390/ijms20215367)
Supplement: Supplementary file 1 [file ijms-20-05367-s001.pdf]

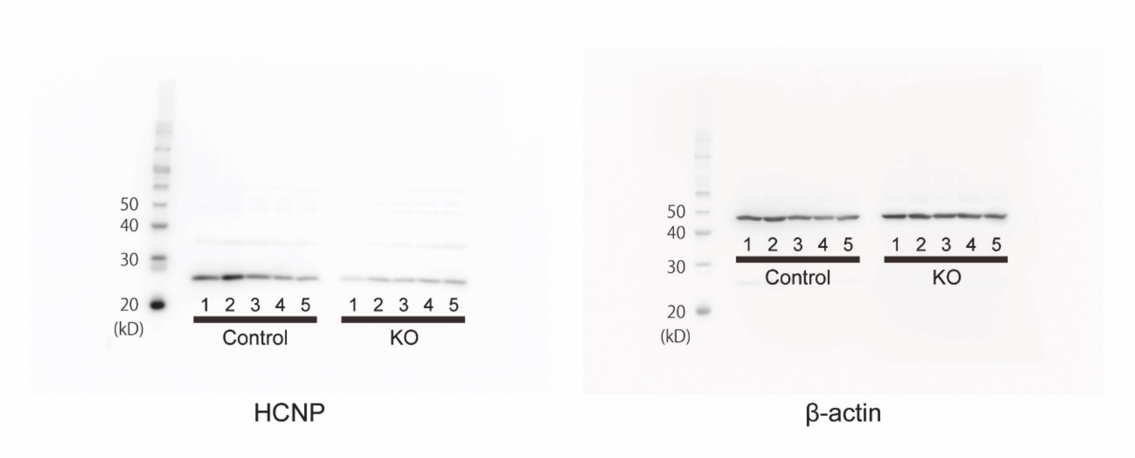

Figure S1. Unprocessed images for Figure 1B (left).

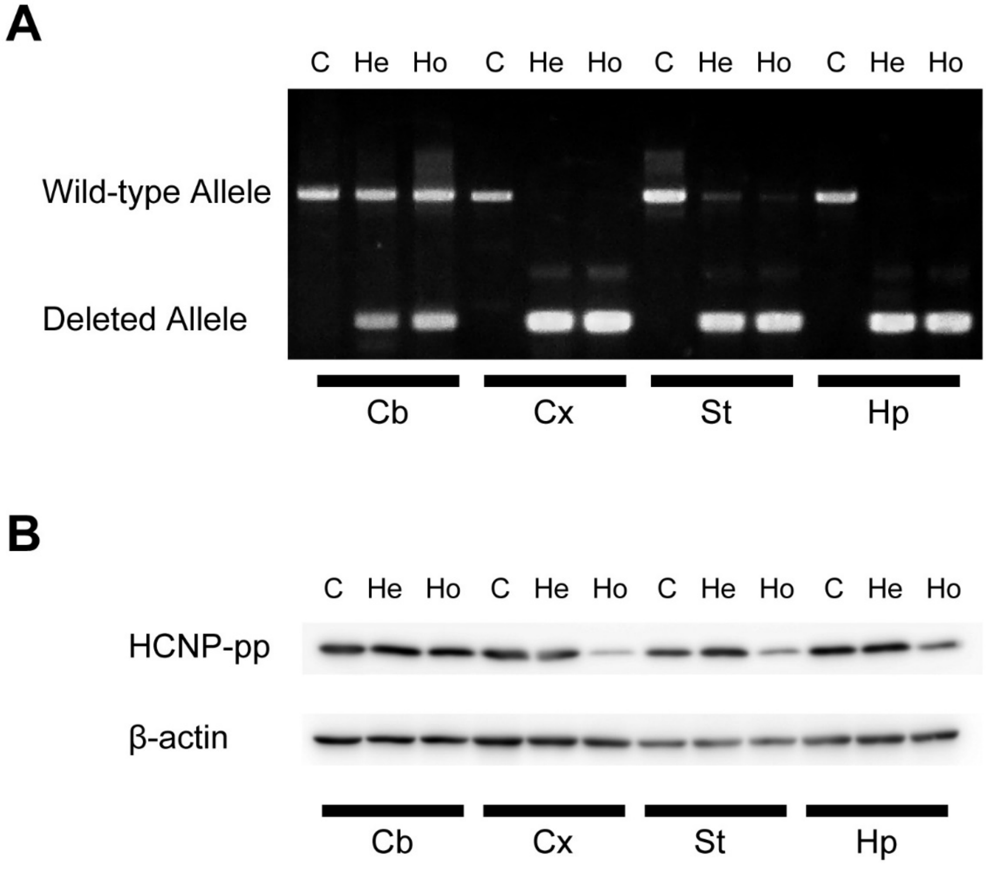

Figure S2. PCR analysis of *HCNP-pp* gene and Western blot analysis of HCNP-pp and β-actin in Control, heterozygous (He) and homozygous (Ho) HCNP-pp KO mice. (**A**) Genomic analysis by PCR. Successful deletion of HCNP-pp genomic DNA was mainly seen in the frontal cortex (Cx), hippocampus (Hp), and striatum (St) in both heterozygous (He) and homozygous (Ho) HCNP-pp KO mice. In the cerebellum (Cb), a part of the HCNP-pp genome was incidentally deleted in both He and Ho HCNP-pp KO mice. In littermate control (C) mice without the *Cre-ERT* gene, no deleted allele was seen. Forward primer; 5′-GGG CTT GTA GTC TAT CAA CAT TCA-3′, Reverse primer; 5′-TTC CGA TCA TAT TCA ATA ACC CTT A-3′ (**B**) Western blot analysis of HCNP-pp and β-actin in Cb, Cx, St, and Hp in Control, He, and Ho. The amount of HCNP-pp was obviously decreased in Cx, St, and Hp in Ho HCNP-pp KO mice.

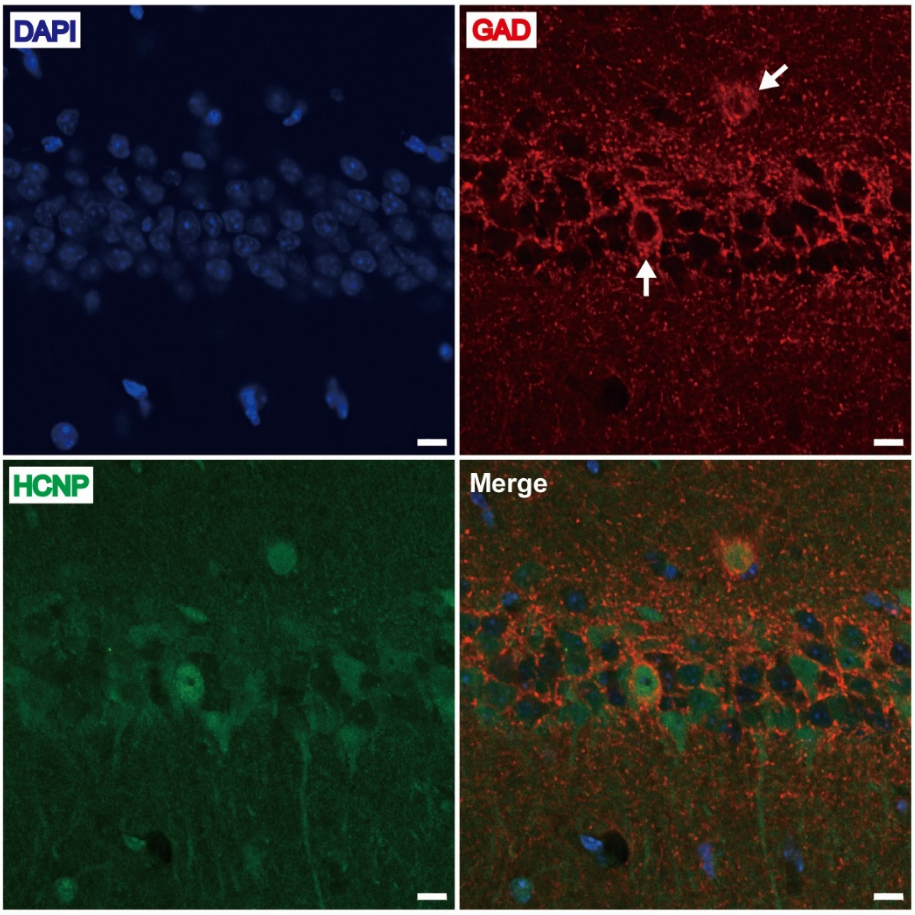

Figure S3. Immunohistochemical staining of the HCNP-pp KO hippocampus (CA1 pyramidal layer) with an anti-HCNP-pp antibody and an anti-glutamic acid decarboxylase (GAD) antibody. GAD-positive neurons (white arrows) show strong HCNP-pp immunoreactivity. Scale bar = 10 μm.
